# Multipack Versus Single-Sterile Implant Supply in Spine Surgery: A Hospital-Based Health Technology Assessment

**DOI:** 10.3390/medicina62071242

**Published:** 2026-06-26

**Authors:** Frederic Bludau, Franz Dally, Johannes Vogel, Sascha Gravius, Joe Mehanna, Viktoria Salopiata, Peter Fennema, Steffen Schulz

**Affiliations:** 1 Department of Orthopaedic and Trauma Surgery, University Medical Centre Mannheim (UMM), 68167 Mannheim, Germany; frederic.bludau@umm.de (F.B.); franz.dally@umm.de (F.D.); johannes.vogel@umm.de (J.V.); sascha.gravius@umm.de (S.G.); viktoria.salopiata@umm.de (V.S.); 2 AMR Advanced Medical Research GmbH, 8078 Männedorf, Switzerland; peter.fennema@amr-cro.com

**Keywords:** spine surgery, pedicle screws, multipack implants, single-sterile implants, Health Technology Assessment, operating-room workflow, packaging waste, implant supply strategy

## Abstract

*Background*: Implant supply strategy in spine surgery affects operative workflow, resource utilization, and packaging-related material use, yet has received limited systematic investigation. This study evaluates single-sterile implants versus multipack implants using a hospital-based Health Technology Assessment (HB-HTA) framework. *Methods*: A non-randomized, mixed-methods comparative study was conducted at a tertiary academic spine center. Time measurements were recorded during eight posterior fusion procedures (four per supply type; n = 18 single-pack screws, n = 20 multipack screws) across three process steps: implant retrieval, sterile transfer, and instrument preparation. Time measurements were recorded per packaging unit; per-implant comparisons were additionally derived for operational interpretation. Packaging volume, weight, and packaging-related CO_2_-equivalent estimates were calculated per implant. Standardized questionnaires were distributed to operating-room (OR) nurses (n = 14/21; 66.7%) and institutional surgeons (n = 11/11; 100%). Manufacturer-provided descriptive process and cost data were analyzed. *Results*: Multipack implants were associated with consistently shorter handling times across all measured process steps. Mean retrieval time per packaging unit was 25.4 s (multipack) versus 58.7 s (single-pack); retrieval time was significantly shorter for multipack units on the Mann–Whitney U test (*p* = 0.004), a result that was robust to supply-related outlier events (*p* = 0.001 after their post hoc exclusion). Packaging-normalized sterile-transfer burden per implant was reduced by a factor of 4.76. Instrument preparation was faster with multipack systems (15.6 s vs. 25.2 s). Packaging volume per implant was reduced by a factor of 5.6, and packaging weight by a factor of 2. Packaging-related CO_2_-equivalent estimates were lower for multipack implants (0.017 kg vs. 0.026 kg per implant). Survey responses indicated predominantly positive evaluations of workflow and handling efficiency. A trade-off was identified regarding the potential disposal of unused implants (noted by 73% of institutional surgeons). Manufacturer-provided descriptive data suggested scale effects in packaging and sterilization processes. *Conclusions*: Under high-volume academic conditions, multipack implants were associated with shorter implant-handling process times, favorable staff perceptions, and reduced packaging-related material burden while introducing trade-offs that require local evaluation. These exploratory findings suggest that the implant supply strategy is an underexplored but potentially relevant dimension of surgical process optimization in spine surgery.

## 1. Introduction

Spine surgery has undergone substantial advances in implant design, navigation, and surgical technique over recent decades. However, operative performance is also influenced by logistical factors such as implant retrieval, verification, and sterile handling [1,2]. These aspects have received limited systematic investigation despite their potential cumulative impact on operative time, resource consumption, and staff workload.

Historically, spinal implants were provided through reusable tray-based systems that offered immediate access to all screw sizes within the sterile field. While efficient in terms of workflow, this approach raised concerns regarding implant contamination during prolonged exposure, surface degradation from repeated reprocessing cycles, and evolving regulatory requirements for traceability [3,4,5,6]. Consequently, single-sterile implants have become standard in many centers, offering guaranteed sterility, full traceability via Unique Device Identifiers (UDI), and clear liability allocation [7,8].

However, single-sterile supply has also introduced new challenges. Each implant requires individual retrieval from storage, packaging verification, sterile transfer, and documentation. In typical posterior fusion procedures, this creates a repetitive sequence of handling steps that may prolong non-surgical process time [9,10,11]. In Germany alone, approximately 40,000 posterior spinal fusions are performed annually, corresponding to an estimated 240,000 pedicle screws per year [12].

Multipack implant systems represent an alternative approach in which multiple implants (e.g., two pedicle screws and two set screws) are bundled into a single-sterile packaging unit. One such system is the Perla™ TL (Spineart, Geneva, Switzerland), introduced in Europe in 2021 [13], which bundles two pedicle screws and two set screws into a single-sterile packaging unit. This bundling may reduce the number of handling steps, decrease packaging volume, and influence logistical processes at both the hospital and manufacturer levels.

The present study evaluates whether this modification in implant supply architecture is associated with measurable differences in intraoperative handling processes, staff perception, packaging-related resource use, and selected manufacturer-reported process-cost parameters. The evaluation uses a hospital-based Health Technology Assessment approach suited to institutional decision-making rather than national-level policy evaluation.

Specifically, we hypothesized that multipack implants (H1) reduce intraoperative process time, (H2) increase satisfaction among operating-room staff and surgeons, (H3) reduce packaging volume and packaging-related material burden, and (H4) decrease selected process costs at the manufacturer level.

## 2. Materials and Methods

### 2.1. Study Design

This study followed a non-randomized, comparative design using a mixed-methods approach. The evaluation was conducted in the second half of 2025 at a university-based tertiary referral center performing a broad spectrum of primary and revision spine surgery, including complex multi-segment procedures. The analysis was structured according to a hospital-based Health Technology Assessment framework, integrating quantitative process measurements with standardized questionnaire data and descriptive manufacturer-level cost information [14,15,16].

### 2.2. Implant Systems

Two pedicle screw systems were evaluated, differing in their supply and packaging strategy:

Single-sterile implants: Everest™ Spinal System (Stryker Corporation, Kalamazoo, MI, USA). Each pedicle screw and each set screw is individually packaged in a separate sterile unit.

Multipack implants: Perla™ TL (Spineart SA, Geneva, Switzerland). Two pedicle screws and two set screws are bundled into a single-sterile packaging unit.

Both systems are in routine clinical use at the study center for the investigated indications. Implant choice followed routine institutional practice and surgeon preference for comparable posterior fusion indications rather than study allocation; the two systems are used interchangeably for these indications at the study center. The evaluation therefore addresses the operational consequences of supply strategy, not clinical implant performance or biomechanical equivalence. The present study evaluates supply strategy; formal biomechanical equivalence testing was not part of this investigation. Accordingly, the comparison reflects real-world supply models embodied in two commercially available systems rather than an experimentally isolated packaging intervention.

### 2.3. Time Measurements (Hypothesis H1)

Prospective time measurements were conducted during eight posterior spinal fusion procedures (four per supply type). A total of 18 single-pack screws and 20 multipack screws were timed. The procedures were selected pragmatically to achieve approximate comparability in procedure type, extent, and complexity.

Time measurements were recorded per packaging unit. Because multipack units contained two pedicle screws, per-implant estimates were additionally derived for operational comparison between systems. Three process steps were recorded independently:

1. Search and retrieval: Time from the surgeon’s request to locating and bringing the correct implant box into the operating room (circulating nurse). For single-sterile implants, this included simultaneous retrieval of the pedicle screw and its corresponding set screw. For multipack implants, this included the entire 4-implant unit.
2. Sterile transfer: Time for transferring the implant(s) from the outer packaging into the sterile field.
3. Instrument preparation: Time for the scrub nurse to connect the implant to the screwdriver.

Outlier events—defined as instances where the requested implant was unavailable in the storage cart, requiring return to the surgeon for an alternative selection—were documented separately and reported both inclusive and exclusive of outlier-adjusted analyses.

### 2.4. Staff Satisfaction Surveys (Hypothesis H2)

Standardized digital questionnaires (SurveyMonkey, SurveryMonkey Inc., San Mateo, CA, USA) were distributed between November 2025 and January 2026 to the following respondent groups:

1. OR nursing staff of the spine-surgery cluster at the study center (21 eligible; 14 responded; 66.7% response rate). All respondents had practical experience with both supply systems.
2. Institutional spine surgeons at the study center (11 contacted; 11 responded; 100%), comprising five neurosurgeons and six orthopedic and trauma surgeons, all of whom reported spine surgery as their main field of activity, with more than eight years of spine-surgery experience and routine use of both supply systems.

Survey domains included workflow efficiency, handling ease, safety perception, environmental impact, and overall preference. Responses were captured on 6-point Likert scales. Each questionnaire combined closed Likert-scaled items across these domains with an optional free-text field. Operating-room logistics staff were not part of the structured survey and contributed contextual operational feedback only.

### 2.5. Packaging-Related Environmental Analysis (Hypothesis H3)

Packaging analysis was performed on ten implants per supply type, assessed immediately after opening in the OR. Measurements included outer carton dimensions and volume (cm^3^), total packaging weight per unit (g), weight of sterile inner packaging (g), and material composition (cardboard, PVC, Tyvek^®^). CO_2_-equivalent estimates were derived from material type and weight using an online emissions calculator (WasteBits, 2026) as a simplified packaging-focused approximation rather than a full life-cycle assessment [17].

### 2.6. Manufacturer-Level Cost Analysis (Hypothesis H4)

Spineart SA provided anonymized process and cost data for both multipack and single-sterile configurations of their own product line. The analysis focused on three domains: packaging, sterilization (gamma irradiation), and logistics. This comparison is descriptive, limited to a single manufacturer, and does not constitute a full health-economic evaluation.

### 2.7. Statistical Analysis

Quantitative data were analyzed using IBM SPSS Statistics Version 30, (IBM Corp., Armonk, NY, USA) and Microsoft Excel Version 16 (Microsoft Corp., Redmond, WA, USA). Descriptive statistics include means, ranges, and standard deviations. Group comparisons for time data were performed using Student’s t-tests. Distributional assumptions were assessed using the Shapiro–Wilk test. Because retrieval times in the single-sterile group departed from normality owing to documented supply-related outlier events, the non-parametric Mann–Whitney U test was used as the primary between-group comparison for time data, with Student’s t-tests reported for reference. Continuous outcomes are summarized as mean ± standard deviation and as median with interquartile range. All *p*-values are two-sided. Survey data are presented descriptively.

## 3. Results

### 3.1. Time Efficiency

#### 3.1.1. Search and Retrieval

Mean search and retrieval time per packaging unit was 58.7 ± 75.3 s for single-sterile implants versus 25.4 ± 11.6 s for multipack units; corresponding medians were 38 s (IQR 31–40) and 24.5 s (IQR 18–28). The large single-sterile standard deviation reflects the supply-related outlier events described below. Three outlier events occurred during the study period (two in the single-pack group at 325 s and 183 s; one in the multipack group at 52 s), each caused by the requested implant not being immediately available in the storage cart.

On the Mann–Whitney U test, the primary between-group comparison, search and retrieval time was significantly shorter for multipack units than for single-sterile implants (*p* = 0.004). Because this test is rank-based, the result was robust to the supply-related outlier events and remained significant after their post hoc exclusion (*p* = 0.001). A two-sample t-test, which is sensitive to the outlier-driven variance in the single-sterile group, did not reach significance on the full dataset (*p* = 0.08) but did so after outlier exclusion (*p* = 0.001). The Shapiro–Wilk test indicated a non-normal retrieval-time distribution in the single-sterile group (*p* < 0.001), supporting the use of the non-parametric test as the primary comparison. The post hoc outlier exclusion was based on a defined external cause (implant unavailability), was not prespecified, and is reported as a sensitivity analysis.

Because each multipack unit contained two pedicle screws, the effective per-screw retrieval time was approximately half the per-unit retrieval time. On this derived basis, retrieval time was approximately 4.6-fold higher for single-sterile than for multipack implants. This difference primarily reflects the smaller number of discrete retrieval events per implant with multipack units (one packaging unit yielding four implants), together with the guaranteed inclusion of matching set screws, which reduced repeat retrieval steps.

#### 3.1.2. Sterile Transfer

**Figure 1:**
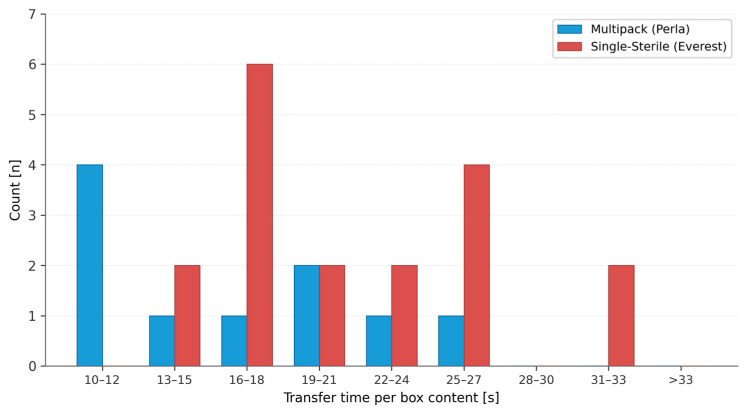
Transfer time into the sterile field by supply type. Note: each multipack unit contains 4 implants.

On a screw-level basis, sterile-transfer time was 20.8 ± 5.1 s for single-sterile implants versus 16.3 ± 5.3 s for multipack implants (Figure 1). This difference was borderline on the Mann–Whitney U test (*p* = 0.052). When normalized to the number of implants delivered per sterile packaging unit, the transfer burden per implant was reduced by a factor of 4.76 with the multipack system. This reflects the reduced number of sterile-transfer events required with bundled packaging.

#### 3.1.3. Instrument Preparation

Mean time for the scrub nurse to connect the implant to the screwdriver was 15.6 s (range 10–20 s) for multipack implants versus 25.2 s (range 18–38 s) for single-sterile implants, corresponding to an approximately 38% shorter preparation time. The reason for this difference was not formally investigated. Standard deviations were 5.2 s for single-sterile and 2.9 s for multipack implants, and the between-group difference was significant on the Mann–Whitney U test (*p* < 0.001).

### 3.2. Staff Satisfaction

#### 3.2.1. OR Nursing Staff

Among the 14 respondents (66.7% response rate), multipack implants were rated favorably across retrieval speed, sterile-transfer ease, scrub nurse handling, waste reduction, and storage requirements. Perceived safety was also rated positively, as the matching number of set screws was guaranteed within each unit. Responses regarding disposal of unused implants were more mixed, suggesting recognition of a potential trade-off rather than a dominant practical problem. Free-text comments highlighted reduced documentation steps and faster overall workflow as key advantages.

#### 3.2.2. Institutional Spine Surgeons

Among the 11 institutional spine surgeons (100% response rate), overall evaluation favored the multipack system (91% agreed advantages outweigh disadvantages), particularly regarding reduced retrieval time and fewer perceived workflow interruptions during longer instrumentation sequences. In free-text responses, one surgeon explicitly described improved procedural continuity during long-segment fusions. Safety perceptions were more heterogeneous (73% positive). Potential disposal of unused implants was viewed critically by 73% of respondents.

#### 3.2.3. Operating-Room Logistics Feedback (Contextual)

Operating-room logistics staff were not part of the structured survey. In informal feedback, they noted perceived advantages regarding storage space and waste reduction, while observing that the local ordering system still required item-level registration irrespective of packaging format—an institutional process limitation independent of the implant system.

### 3.3. Packaging Analysis

**Figure 2:**
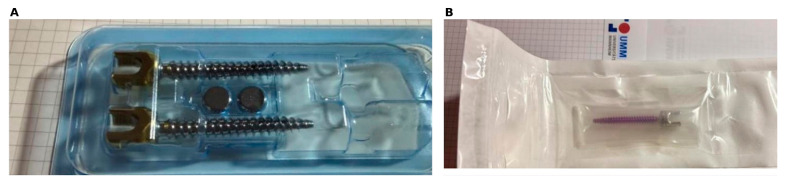
Direct comparison of sterile inner packaging. (**A**) Multipack. (**B**) Single-sterile.

Single-sterile implants required 5.6 times the packaging volume per implant compared to multipack units (425 mL vs. 76.5 mL per implant). Total packaging weight per implant was reduced by a factor of 2 (Table 1). The sterile inner packaging differed in design: multipack implants used a PVC blister with a Tyvek^®^ lid, while single-sterile implants used a PVC pouch with double Tyvek^®^ heat-sealed envelopes (Figure 2). CO_2_ equivalents per implant were 0.026 kg for single-sterile and 0.017 kg for multipack packaging, corresponding to an approximately 35% lower packaging-related CO_2_ estimate for the multipack configuration under the assumptions of the simplified calculation model.

### 3.4. Manufacturer-Level Effects

Within the manufacturer-reported process model, packaging and sterilization costs per implant approximately halved with the multipack configuration (from 4.50 CHF per screw-and-set-screw pair in single-sterile format to 2.25 CHF per equivalent in multipack format). Since sterilization by gamma irradiation is charged per volume, the bundling of four implants into one unit of equivalent volume produced a proportional cost reduction. These data derive from a single manufacturer source and are reported descriptively. 

### 3.5. Summary of Findings

The main findings across all evaluaton dimensions are summarised in Table 2.

**Table 2:** Summary of findings across evaluation dimensions.

Dimension	Key Finding	Direction
Time: retrieval	4.6× shorter retrieval time per screw (multipack)	Favors multipack
Time: sterile transfer	Packaging-normalized sterile-transfer burden reduced 4.76×	Favors multipack
Time: preparation	38% reduction (15.6 s vs. 25.2 s)	Favors multipack
Staff satisfaction	Predominantly positive; 91% of institutional surgeons reported net advantage	Favors multipack
Packaging volume	5.6× reduction per implant	Favors multipack
Packaging weight	2.0× reduction per implant	Favors multipack
Packaging CO_2_e per implant	35% reduction (packaging-based estimate)	Favors multipack
Manufacturer costs	~50% lower packaging/sterilization process cost per implant (single source)	Favors multipack
Implant disposal	Identified as trade-off (73% critical)	Favors single-sterile

## 4. Discussion

This study provides a multidimensional comparison of two implant supply strategies in spine surgery, evaluated within a hospital-based Health Technology Assessment framework. The findings suggest directionally favorable operational signals for the multipack approach across temporal, perceptual, packaging-related, and descriptive process-cost dimensions, while identifying a relevant trade-off regarding potential implant disposal.

### 4.1. Temporal Effects

The observed handling-time reductions with multipack implants were consistent across all three measured process steps. Previous studies in hand and ankle surgery have documented that single-sterile implant handling adds approximately 96 s per screw compared to tray-based systems [9,10,11]. In the present study, the effective per-screw time reduction achieved through multipack bundling was approximately 61 s, suggesting that a substantial proportion of the workflow penalty associated with single-sterile handling may be recoverable through bundling strategies.

**Figure 3:**
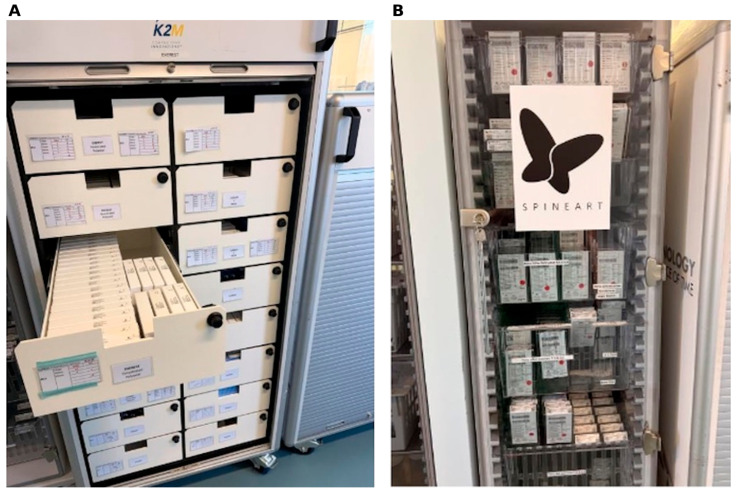
Storage configuration. (**A**) Two module carts for single-sterile. (**B**) Single cart for multipack.

The statistical analysis warrants careful interpretation. On the rank-based Mann–Whitney U test, the retrieval-time difference was significant on the full dataset (*p* = 0.004) and remained significant after post hoc exclusion of the three supply-related outlier events (*p* = 0.001); a two-sample t-test reached significance only after outlier exclusion, reflecting its sensitivity to the outlier-driven variance in the single-sterile group. The outlier events themselves may be operationally relevant, as larger single-unit inventories can increase storage complexity and the opportunity for retrieval failure. The single-sterile storage configuration required two full module carts, whereas the multipack system required only one under the storage layout in place at the study center (Figure 3). Both supply systems were stored in the same type of cabinet, positioned identically relative to the operating room and the circulating nurse, and held a comparable range of screw sizes and types, so the retrieval pathway was equivalent for the two systems. The requirement for two single-sterile carts reflected the substantially larger physical volume of the individually packaged implants rather than poorer organization. Because this difference stems from packaging dimensions, optimized storage is unlikely to substantially reduce it.

Regardless of the statistical question, the consistency of directional findings across all three process steps and across multiple procedures is perhaps more informative than any single *p*-value. In the context of a high-volume center with substantial fixed operating-room costs [18,19], even modest per-implant time savings may become operationally relevant when accumulated across a high annual case volume [20].

### 4.2. Staff Perception and Workflow Continuity

Survey responses indicated a preference for multipack implants across both respondent groups, with varying degrees of agreement. The high response rates among OR nursing staff (66.7%) and institutional surgeons (100%) suggest that the supply strategy is perceived as a topic of practical relevance in daily clinical work.

Particularly notable was the emphasis on reduced workflow interruptions, especially during longer-segment instrumentations. One institutional surgeon explicitly described improved procedural continuity during extended fusions. This observation is consistent with the literature on intraoperative disruptions and surgical human factors, where interruptions in procedural rhythm have been associated with increased cognitive demands and reduced operative efficiency [21,22,23]. This interpretation should be considered cautiously, however, as cognitive load and flow states were not directly measured in the present study. The convergence of quantitative time reductions and subjective reports of smoother workflow is consistent with a process-level benefit, but further investigation with validated instruments would be needed to confirm this.

The repeated concern regarding the disposal of unused implants merits attention. Because multipack units contain two pedicle screws of identical size, an odd number of same-size screws or a change in intraoperative sizing creates surplus implants that cannot be re-sterilized. This represents a genuine trade-off between workflow efficiency and material waste—a tension also identified in the broader literature on surgical implant sustainability [24,25]. The frequency and economic impact of such disposal events were not systematically quantified in this study and warrants dedicated investigation.

### 4.3. Packaging-Related Environmental Considerations

The packaging analysis demonstrated substantial volume and weight reductions per implant with multipack systems. Given the substantial waste generated in operating rooms [26], even incremental packaging reductions may be relevant when scaled across high-volume procedures.

The CO_2_-equivalent calculation should be interpreted as a packaging-focused approximation. A comprehensive life-cycle assessment including production, transport, and disposal pathways was not performed [27]. Importantly, the production-related carbon footprint of a titanium implant is likely to be materially larger than the packaging-related difference quantified here. If a multipack unit leaves one pedicle screw and set screw unused and these are discarded, the embodied production footprint of the wasted implant could offset or exceed the packaging-related CO_2_ advantage [28]. The packaging-related saving is also small relative to the total carbon footprint of a surgical procedure [29]. The net environmental balance therefore cannot be determined without a formal life-cycle assessment incorporating implant-waste frequency and implant production footprints [24]; the present analysis is confined to the packaging dimension.

### 4.4. Manufacturer-Level Perspective

The manufacturer’s data indicate that bundling implants into multipack units produces scale efficiencies in packaging and sterilization. Since both processes are volume-based, the cost per implant decreases proportionally with increased implant density per unit. These findings are limited to one manufacturer and do not allow conclusions about system-level health economics. They do, however, suggest that supply strategy modifications may have effects beyond the hospital level, particularly regarding packaging and sterilization processes—an aspect relevant for hospital–industry procurement discussions but one that should not be overinterpreted based on single-source descriptive data. In particular, these descriptive packaging and sterilization figures do not capture implant acquisition costs, the cost of any unused implants discarded from multipack units, staff time, operating-room opportunity costs, storage logistics, or institution-specific procurement prices; a formal economic evaluation would need to incorporate these components. Institution-specific implant acquisition costs were confidential and could not be disclosed; the two systems did not, however, differ materially in acquisition cost. The economic impact of discarded unused implants could therefore not be quantified in this study.

### 4.5. Multidimensional Assessment

The hospital-based HTA framework proved useful for structuring the evaluation across multiple dimensions that would not be captured by a single-outcome clinical study. The convergence of findings across temporal, perceptual, packaging-related, and descriptive process-cost dimensions—all directionally favoring the multipack approach, albeit with varying effect sizes and levels of evidence—provides a broader basis for institutional decision-making than any single metric alone [14,15].

Importantly, the evaluation also revealed persistent trade-offs. The potential for implant waste, the current limitations of hospital ordering systems, and the dependence on a single manufacturer offering multipack pedicle screws all represent factors that may influence adoption decisions. The HTA approach makes these tensions explicit rather than reducing the assessment to a single favorable outcome measure.

## 5. Limitations

Several limitations should be considered when interpreting these results. The study was conducted at a single university center with high case volume and specialized logistics infrastructure, which limits generalizability to other settings. The study design was non-randomized, and the sample size for time measurements was small (n = 38 screws across 8 procedures). Confounding due to pragmatic procedure selection cannot be excluded. Statistical findings should be interpreted as exploratory and hypothesis-generating rather than confirmatory, with the non-parametric Mann–Whitney U comparison treated as primary and the outlier-excluded analysis as a sensitivity analysis. Because multiple screws were timed within each of the eight procedures, the screw-level observations are not fully independent and were treated as such; this clustering is a further reason to read the time comparisons as indicative rather than definitive.

Because the compared systems were sourced from different manufacturers, the observed differences cannot be attributed exclusively to packaging architecture; product-specific design and labeling features may also have contributed. The comparison reflects real-world supply models rather than an experimentally controlled packaging intervention.

The environmental analysis was limited to packaging and did not include a full life-cycle assessment. The CO_2_-equivalent estimates are simplified approximations. The manufacturer cost data derive from a single source. The institutional surgeon survey, while showing a high response rate, represents a convenience sample and is not representative of all spine surgical practice.

Finally, the rate and clinical or economic impact of implant disposal from multipack units were not systematically quantified and warrant specific investigation in future studies.

## 6. Conclusions

Under the conditions of a high-volume academic spine center, multipack implant systems were associated with shorter intraoperative handling times, favorable staff perceptions, lower packaging-related material burden, and lower manufacturer-reported process costs compared with single-sterile implants. These exploratory findings are best regarded as hypothesis-generating operational signals rather than confirmatory evidence of superiority, and indicate that implant supply strategy is a relevant and understudied aspect of implant-supply organization with implications for workflow and packaging-related resource use in spine surgery.

Adoption decisions should account for local case volume, logistics infrastructure, and the identified trade-off regarding potential implant waste. Multicenter studies with larger sample sizes and formal cost-effectiveness analyses would strengthen the evidence base for supply strategy decisions in spine surgery.

## Figures and Tables

**Table 1:** Packaging and implant weight comparison per supply type.

Parameter	Single-Sterile	Multipack	MP Per Implant	Factor
Packaging volume (mL)	425	306	76.5	5.6×
Total weight per unit (g)	43.24	86.89	21.72	2.0×
Carton + UDI label (g)	22.33	53.3	13.33	1.7×
Sterile packaging (g)	10.08	14.69	3.67	2.7×
Packaging CO_2_e per implant (kg)	0.026	—	0.017	1.5×

Single-sterile unit = 1 pedicle screw + 1 set screw. Multipack unit = 2 pedicle screws + 2 set screws. Factor = ratio of single-sterile to multipack per-implant values. CO_2_e: simplified packaging-focused approximation [17].

## Data Availability

De-identified study data are available from the corresponding author upon reasonable request, subject to institutional and legal constraints.
